# The effects of dietary instruction on cardiovascular risk markers after smoking cessation: study protocol for a multicenter randomized controlled trial in Japan

**DOI:** 10.1186/s13063-018-2919-6

**Published:** 2018-10-04

**Authors:** Maki Komiyama, Yuka Ozaki, Hiromichi Wada, Hajime Yamakage, Noriko Satoh-Asahara, Tatsuya Morimoto, Akira Shimatsu, Yuko Takahashi, Koji Hasegawa

**Affiliations:** 1 Clinical Research Institute, National Hospital Organization Kyoto Medical Center, 1-1 Mukaihata-cho, Fukakusa, Fushimi-ku, Kyoto, 612-8555 Japan; 2 Division of Molecular Medicine, School of Pharmaceutical Sciences, University of Shizuoka, Shizuoka City, Japan

**Keywords:** Smoking cessation, Weight gain, Obesity, Nutritional guidance, Cardiovascular risk, Adiponectin

## Abstract

**Background:**

Weight gain frequently occurs after smoking cessation (SC); the risk of new-onset diabetes mellitus increases for several years after SC. However, no large-scale, randomized controlled trials have examined the effects of nutritional guidance on post-SC cardiovascular risk. The current trial will enroll individuals who successfully quit smoking with the help of a SC clinic and who gain weight, to determine the effects of nutritional guidance on cardiovascular, glucose, and lipid metabolism biomarkers.

**Methods/design:**

This is a multicenter, prospective, parallel-group, randomized controlled trial. Some 250 individuals who successfully quit smoking with the help of a SC clinic and who gain weight (an increase of ≥ 1.25% Body Mass Index (BMI) between the first and the fifth visit to the SC clinic) will be enrolled within 1 month of the final (fifth) visit to the SC clinic. These participants will be randomly assigned to an intervention group (125 individuals receiving nutritional guidance) or a control group (125 individuals not receiving nutritional guidance). A registered dietitian will provide nutritional guidance once every 3 months for a total of three sessions. The primary endpoint for this trial will be the level of adiponectin, a predictor of cardiovascular risk that reflects weight and smoking status. Secondary endpoints will be levels of cardiovascular, glucose, and lipid metabolism biomarkers, BMI, abdominal circumference, and the percentage of individuals who quit smoking for a prolonged period.

**Discussion:**

This trial will determine the benefits of nutritional guidance with respect to post-SC weight gain. The findings should provide useful information for devising a quality protocol for SC education to prevent cardiovascular disease.

**Trial registration:**

The study is registered at the University Hospital Medical Information Network Clinical Trials Registry (UMIN000030282). Registered on 6 December 2017.

**Electronic supplementary material:**

The online version of this article (10.1186/s13063-018-2919-6) contains supplementary material, which is available to authorized users.

## Background

### Effects of smoking

Smoking contributes to the development of fatal cardiovascular diseases and malignancies. Worldwide, approximately 5.4 million people die from preventable smoking-related diseases each year; this number corresponds to 10% of all adults [1]. According to the Ministry of Health, Labor, and Welfare, heart disease is the second-leading cause of death in Japan, accounting for approximately one sixth of Japanese deaths [2]. National medical expenditures have exceeded 38 trillion yen, accounting for more than 10% of the national income. The greatest portion (21%), 5.8 trillion yen, is spent on cardiovascular disease. The Japan Collaborative Cohort Study involved 95,000 Japanese, and it found that smoking increased the risk of death from cardiovascular disease 1.6–2.0-fold, resulting in exorbitant medical expenditures [3]. Nonetheless, 30% of male adults and 10% of female adults in Japan smoke [4]. Japan has one of the highest smoking rates among developed countries, and smoking cessation (SC) must be promoted further.

### Weight gain after smoking cessation

Efforts to curb smoking should significantly decrease the development of cardiovascular disease and sharply curtail medical expenditures. However, individuals are known to gain weight after SC, making obesity prevention and subsequent medical expenditures a concern [5, 6]. The extent of weight gain varies depending on the study, but men who quit smoking on their own gain 2.8 kg on average, whereas women gain an average of 3.8 kg; 20–40% of people gain 13 kg or more [6]. Weight gain after SC can cause individuals to resume smoking, which increases cardiovascular risk. SC aids, such as nicotine patches or varenicline, help minimize weight gain after SC; nevertheless, individuals can gain weight for several years after SC despite receiving treatment. Weight gain frequently results in poorer glucose tolerance and a resumption of smoking. We have reported a case in which glucose tolerance significantly decreased with weight gain after SC [7]. The current authors have previously examined predictors associated with weight gain during SC treatment. Smokers with a high Fagerström Test for Nicotine Dependence (FTND) score upon an initial visit to a SC clinic (smokers with a high level of nicotine dependence) were more likely to gain weight after quitting smoking [8]. The advantages of SC are intricately combined with the disadvantages of post-SC weight gain. α1-antitrypsin low-density lipoprotein (AT-LDL) complex is an oxidatively modified LDL that promotes atherosclerosis. We had previously found that the level of AT-LDL in the blood is closely related to smoking status, that AT-LDL levels improve (decrease) 3 months after SC [9], and that cardiovascular markers do not improve in individuals who gain a substantial amount of weight after quitting smoking [10]. These findings suggest that post-SC weight gain might increase the risk cardiovascular events even though the individual quits smoking. This hypothesis is compatible with a study reporting no clear advantages of SC in diabetics who gain 5 kg or more [11]. In other words, preventing weight gain after quitting smoking could further reduce cardiovascular risk. Smoking and obesity are closely related, and they synergistically increase cardiovascular risk factors such as cerebral infarction and myocardial infarction. Promptly addressing this problem is vital in terms of medical and social costs.

### Standard smoking cessation treatment in Japan

SC treatment will be conducted according to the Standard Procedures for Anti-Smoking Treatment (originally issued in March 2006 by the Japanese Circulation Society, the Japan Lung Cancer Society, and the Japanese Cancer Association) [12]. SC treatment consists of using SC aids, such as nicotine patches or varenicline, along with nondrug therapy such as counseling from a physician or nurse. Smokers may continue to gain weight for 2–3 years after SC. However, there are no specific practice guidelines regarding minimizing weight gain after completing the 12-week SC treatment covered by the National Health Insurance. The standard procedures for anti-smoking treatment list weight gain as a possibility despite the use of SC aids; however, it does not list specific methods to deal with problem.

### Nutritional guidance for individuals who successfully quit smoking

Although diet is understood to affect obesity, appropriate dietary intake and a balanced diet for each individual are unclear, hampering obesity alleviation. Nutritional guidance allows a registered dietitian to review an individual’s diet in light of their energy expenditure and nutritional balance. It also helps the dietitian and individual to devise obesity prevention strategies and ameliorate illness, which has a substantial impact on medical economics. A study has reported that providing nutritional guidance to women who resume smoking due to fear of gaining weight helps inhibit post-SC obesity and increases the percentage of women who quit smoking for a prolonged period [13]. However, large-scale prospective, comparative trials have not yielded evidence for the effects of nutritional guidance on cardiovascular risk for every individual who is likely to gain weight after SC.

### Objective of this trial

The aim of this trial is to determine and compare the effectiveness with which nutritional guidance prevents obesity after SC, the effects of nutritional guidance on cardiovascular, glucose, and lipid metabolism biomarkers, and the effects of nutritional guidance on individuals who quit smoking for a prolonged period. This trial will involve individuals who successfully quit smoking with the help of a SC clinic and gained weight.

Inhibiting weight gain after SC will help maintain glucose tolerance and prevent new-onset diabetes mellitus. An altered body composition with increased visceral fat reduces glucose tolerance while promoting oxidative stress and inflammation. However, a previous study reported that weight gain due to SC increases not only fat but also bone and muscle tissues [14]. Adiponectin levels are closely and inversely correlated with the amount of visceral fat, and numerous studies have reported that decreased adiponectin levels are associated with decreased glucose tolerance. Furthermore, adiponectin levels decrease even when weight gain is inhibited by resuming smoking. Among the numerous markers and items studied, blood adiponectin levels are particularly closely associated with abdominal obesity, smoking, oxidative stress, and obesity- and smoking-associated inflammation. Thus, adiponectin is an optimal marker for use in our study, and we have selected it as the primary endpoint.

## Methods/design

### Design and sample size

This trial will be a multicenter, prospective, parallel-group randomized controlled trial. Participants will be assigned either to a group receiving nutritional guidance (once every 3 months) or to a group not receiving nutritional guidance. We chose 3-monthly visits because nutritional guidance typically continues for about 6 months. In addition, a previous study reported that frequent visits have a greater effect on weight gain and that follow-up is needed at least once every 3 months [15]. Participants will be enrolled for 3 years after the trial is approved by the Central Ethics Review Committee. The total duration of the trial will be 6 years (scheduled duration: April 2016 to March 2022).

Our target enrollment is 250 participants (125 individuals in the control group, 125 individuals in the intervention group). No similar clinical studies exist to serve as a reference. Although the exact sample size cannot be calculated, it has been estimated as follows:

In previous studies by our group, changes in adiponectin levels had served as the primary endpoint, and the standard deviation (SD) for changes in adiponectin levels (defined as the percentage of change in adiponectin levels before and after treatment, with the adiponectin level prior to treatment representing 100%) was 20%. We envision a similar approach for the current trial, and the SD is set at 20% to compare the percentage of change in adiponectin levels in the two groups (individuals who do not receive nutritional guidance vs. individuals who do). In order to detect a 10% difference between the assigned groups (effect size: 0.5) at a two-tailed alpha level of 5%, with a power of 80% and an adjustment for multiple primary endpoints, each group will need to consist of 82 individuals. Assuming a 25% drop-out rate during follow-up, based on data amassed from the SC clinic, 110 participants would need to be enrolled in each group. In light of the aforementioned estimates and other factors for error, the required enrollment would ultimately be a total of 250 individuals who visit the SC clinic, or approximately 125 individuals in each group.

The study’s inclusion and exclusion criteria are outlined in Table 1. The participants must meet all of the inclusion criteria and none of the exclusion criteria.

**Table 1:** Inclusion and exclusion criteria

Inclusion criteria	
1. Individuals who successfully quit smoking* upon their fifth visit to the SC clinic;	
2. Within 1 month of an individual’s fifth visit to the SC clinic for SC treatment based on the standard protocol	
3. Individuals with ≥ 1.25% increase in BMI between their first and fifth visits to the SC clinic	
4. Individuals from 20 to 80 years of age when consent is obtained	
5. Individuals from whom consent has been obtained in writing	
Exclusion criteria	
1. Individuals regularly receiving nutritional guidance	
2. Individuals unlikely to benefit from nutritional guidance because of their general condition	
3. Individuals in shock, with a severe infection, in the acute stage of cerebral infarction or myocardial infarction, or with terminal-stage cancer	
4. Individuals with a BMI less than 18.5 (underweight)	
5. Individuals who are pregnant or nursing	
6. Individuals who are regularly receiving corticosteroids	
7. Individuals with poorly controlled diabetes mellitus (i.e., individuals with a HbA1c level consistently above 10.0% despite treatment)	
8. Individuals otherwise deemed ineligible by an investigator for participation in this trial (e.g., individuals with severe dementia or a poorly controlled psychiatric disorder) for reasons of participant safety or to ensure that the trial is conducted openly and fairly	

*BMI* Body Mass Index, *HbA1c* glycosylated hemoglobin, *SC* smoking cessation

* individuals who indicate in an interview that they have not smoked in the past week and have an exhaled carbon monoxide concentration of 7 ppm or lower

### Discontinuation criteria

The trial treatment will be discontinued for participants in any of the following situations:

(1) a participant asks to withdraw their consent; (2) a participant has asked to stop receiving the trial treatment; (3) a participant’s underlying illness is exacerbated or recurs; (4) a participant’s underlying illness recurs or new cancerous lesions are noted; (5) a participant fails to make a visit as a result of a change in address or similar; (6) a participant dies; or (7) a participant is deemed ineligible for enrollment.

Participants who cease receiving the trial treatment will be followed and will receive routine care as drop-outs.

### Ethics approval

All procedures will be performed in accordance with the ethical standards of the Institutional and National Research Committees as well as the 1964 Helsinki Declaration and its later amendments or comparable ethical standards.

### Study protocol

**Fig. 1:**
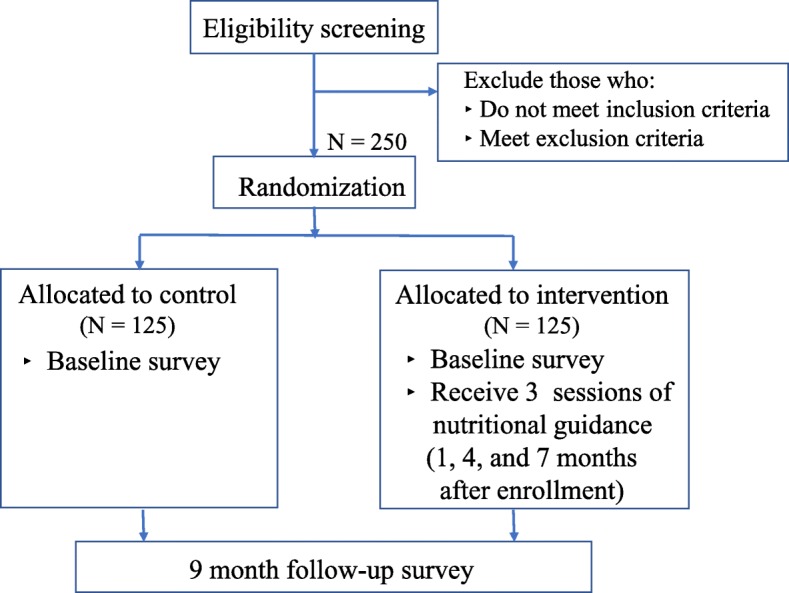
Overview of the study protocol

**Fig. 2:**
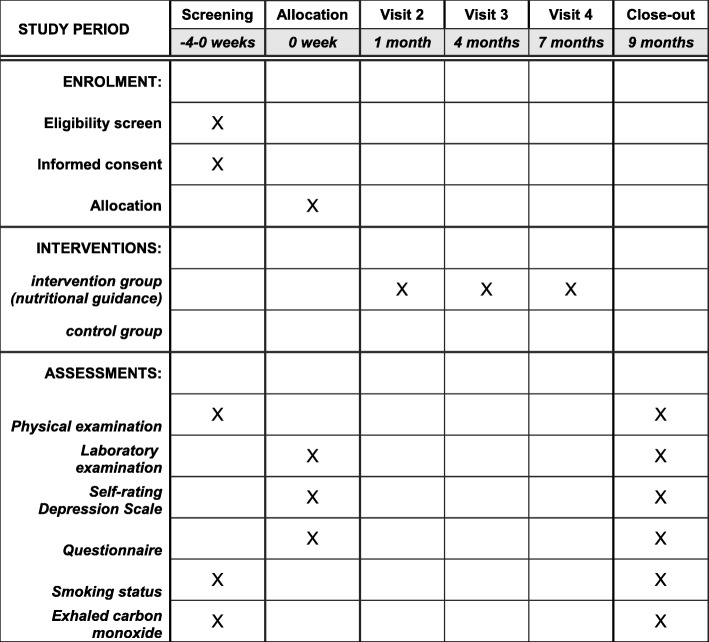
Schedule of enrollment, interventions, and assessments

An overview of the proposed study protocol can be found in Fig. 1. The schedule of enrollment, interventions, and assessments is shown in Fig. 2. The Standard Protocol Items: Recommendations for Interventional Trials (SPIRIT) Checklist is provided in Additional file 1.

### Randomization

Prior to beginning this trial, the University Hospital Medical Information Network’s Internet Data and Information Center for Medical Research will be asked to create a central assignment system using the Electronic Data Capture (EDC) system. Once written consent is obtained from an individual who meets the inclusion criteria, and a lead investigator has verified that the individual meets all of the eligibility criteria and none of the exclusion criteria, the participant’s information will be registered in the EDC system. After registration, the EDC system will assign patient numbers that do not include the participant’s personal information. Immediately after entry, the EDC system will randomly assign the participants to one of two groups (the group receiving nutritional guidance or the group not receiving nutritional guidance) via dynamic allocation. A lead investigator (or study chair) and the study office of the Kyoto Medical Center will be notified of the patient numbers and the group assignments. Based on the EDC system assignment, a lead investigator will schedule nutritional guidance for individuals receiving nutritional guidance and a visit 9 months later for individuals not receiving nutritional guidance. Registration in the EDC system allows adjustments for confounding factors (age, sex, the FTND score, weight gain (kg) before and after SC, and the fear of gaining weight according to a survey) between groups. The participants will be randomized by minimization (a method of dynamic allocation).

### Interventions

The individuals in the experimental group will receive nutritional guidance from a registered dietitian at 1, 4, and 7 months after enrollment. A study has reported that nutritional guidance for 6 months or longer, or in numerous sessions, is effective for preventing weight gain and for discouraging former smokers from resuming smoking [15]. An investigator or associate will explain the significance of the nutritional guidance in this trial (preventing weight gain after SC) to a registered dietitian at each study site. Typically, food intake and the number of meals increase in response to oral cravings and increased appetite after quitting smoking. During nutritional guidance, participants will be educated on the significance of actively managing nutrition while quitting smoking. Participants’ height and weight will be determined, and they will be informed of their optimal weight based on a reasonable Body Mass Index (BMI) (22). An instructional plan to meet an individual’s estimated energy requirements and nutrient intake will be formulated based on the Dietary Reference Intake for Japanese (2015) as stipulated by the Ministry of Health, Labor, and Welfare [16]. In principle, the estimated energy requirement and nutrient intake are based on Article 20, Point 2 of the Health Promotion Act (Act No. 103 of 2002) [16]. The Dietary Reference Intakes help calculate the estimated energy requirement based on basal metabolism, physical activity level, sex, age, height, and weight. It also assesses the status of food intake, whereby changes in weight are used to assess whether the actual energy requirement is lower or higher than the estimated value. If a participant has an illness, such as diabetes, hypertension, dyslipidemia, atherosclerotic disease, or chronic kidney disease, an instructional plan will be devised based on management guidelines for that particular illness. For this, an individual’s energy needs will be determined and their dietary intake assessed so that the individual can be instructed on how to improve their diet.

During follow-up, the participants will log their dietary intake, activity level, and weight to the greatest extent possible. During follow-up, an excess or lack of energy will be assessed in light of weight changes. Targets will be reset while referring to individual logs as needed, and the participants will be encouraged to appropriately manage their energy and nutrient intake and weight. Individuals who resume smoking while receiving nutritional guidance will not be prompted to quit smoking. Items covered and materials used in nutritional guidance will be standardized among the study sites.

### Control conditions

Individuals not receiving nutritional guidance will have a follow-up visit 9 months after enrollment.

### Both groups

Studies have reported that oral antidiabetic, antihypertensive, antidyslipidemic, antipsychotic, and antiepileptic drugs, and other medications can affect weight and adiponectin levels (the primary endpoint of this trial). Thus, the baseline dosage and concomitant medications will, in principle, not be modified, nor will new medications be added during the follow-up of either group. If the baseline dosage or concomitant medications must be modified, or a new medication must be added, the reason for the change, the name of the medication, the dosage, and the duration of administration will be described in detail on the questionnaire. If a participant fails to make a scheduled visit, the individual will be contacted by telephone to ascertain the reason. Upon the conclusion of the trial treatment period, routine treatment covered by National Health Insurance will be provided as needed at the physician’s discretion.

### Items noted and studied

The following items will be assessed during the fifth visit to the SC clinic (upon enrollment) and at 9 months after enrollment:

#### Items that will only be assessed upon enrollment

Height; the number of cigarettes smoked as of the first visit to the SC clinic; the number of years of smoking; nicotine dependence (FTND score); weight gained (kg) between the first and the fifth visit to the SC clinic; the fear of gaining weight according to a survey; and the stage of behavioral change in terms of diet. A survey will be administered on paper at the start of enrollment to assess (1) the fear of gaining weight and (2) the stage of behavioral change with respect to an improved diet in order to prevent weight gain. Responses to both questions will be on a 4-point scale.

#### Items measured upon enrollment and 9 months after enrollment

Items that will be measured as part of treatment covered by National Health Insurance are medication information: (1) oral hypoglycemic drugs, (2) antihypertensive drugs, (3) antidyslipidemic drugs, (4) antipsychotic/antiepileptic drugs, and (5) the name of, and daily dose of, other medications, body composition (weight, BMI, and abdominal circumference); blood pressure and pulse rate; the concentration of exhaled carbon monoxide (CO); a self-rating depression scale; routine biochemistry: lipid profile (total cholesterol, triglycerides, high-density lipoprotein cholesterol (HDL-C), low-density lipoprotein cholesterol (LDL-C)); blood glucose; glycosylated hemoglobin (hemoglobin A1c; HbA1c); kidney function (creatinine, blood urea nitrogen, uric acid, sodium, potassium, and chloride); liver function (total protein, albumin, glutamic oxaloacetic transaminase, glutamic pyruvic transaminase, and gamma-glutamyl transferase); and blood counts: white blood cell count and appearance, red blood cell count, hematocrit, hemoglobin, and platelet count.

Items that will be measured as part of treatment but that are not covered by National Health Insurance are: high-sensitivity C-reactive protein (hsCRP) levels, oxidized LDL complexes (AT-LDL and serum amyloid A-LDL (SAA-LDL)), the LOX Index, adiponectin, and leptin.

After a participant has remained in a seated position for 5 min or longer, their blood pressure will be measured with a sphygmomanometer while keeping the heart and the measurement site (brachial artery) at the same height. Then, 20 mL of venous blood will be collected from the forearm; 10 mL of that blood will be used to perform routine biochemistry and to determine the blood counts. The remaining 10 mL will be centrifuged. A portion of this remaining blood will be refrigerated and used to measure levels of AT-LDL and SAA-LDL, and the rest will be stored in a deep freezer at − 80 °C. The cryopreserved portion will be used to measure levels of hsCRP, the LOX Index, adiponectin, and leptin.

### Observation and testing schedule

#### Primary endpoint

The primary endpoint for this trial will be the adiponectin level. Adiponectin is a peptide hormone secreted by adipocytes that inhibits atherosclerosis [17, 18]. Adiponectin is also a cardiovascular disease risk marker closely related to smoking, obesity, and atherosclerotic disease (i.e., adiponectin levels decrease as a result of smoking or obesity) [17, 18].

#### Secondary endpoints

Secondary endpoints will be the following: (1) levels of oxidized LDL complexes (AT-LDL and SAA-LDL), levels of hsCRP, and the LOX Index; (2) markers of glucose and lipid metabolism: HbA1c, LDL-C, HDL-C, nonHDL-C, and leptin levels; (3) BMI and abdominal circumference; and (4) the percentage of individuals who quit smoking for a prolonged period.

The cardiovascular risk biomarkers serving as secondary endpoints are closely associated with smoking, obesity, and atherosclerotic disease. Oxidatively modified LDL complexes (AT-LDL and SAA-LDL) promote atherosclerosis [9, 10]; AT-LDL is related to smoking status and obesity. A study has suggested that weight gain after quitting smoking might inhibit a decrease (improvement) in AT-LDL after quitting smoking [9, 10, 19]. SAA-LDL is also related to obesity and smoking [20]. Another study has suggested that SAA-LDL might be superior to hsCRP as a cardiovascular marker [21]. High-sensitivity CRP increases as a result of diabetes, obesity, smoking, or age, and hsCRP is related to atherosclerotic disease. The LOX Index is determined by multiplying the level of soluble lectin-type oxidized LDL receptor 1 (LOX-1) (an oxidized LDL receptor found in vascular endothelial cells) by the level of ligand containing ApoB (a modified LDL that binds to LOX-1). A high LOX Index is significantly related to a risk of developing cardiovascular disease, and a low LOX Index is related to a reduced risk of cerebral infarction [22]. Changes in other laboratory results from normal values could serve as the latest indicators with which to assess the progression of atherosclerosis in the future and to assess the risk of cerebral infarction or myocardial infarction in earlier stages [22]. Leptin is a peptide secreted by adipocytes that is involved in inhibiting obesity, given it has action to suppress the appetite and increase energy expenditure.

### Statistical analysis

#### Analysis set

The assigned groups, i.e., the individuals receiving nutritional guidance vs. the individuals not receiving nutritional guidance, will be compared based on the intention-to-treat principle. All data, such as levels of cardiovascular biomarkers and weight obtained from each group upon enrollment and at 9 months after enrollment, will be analyzed. Only individuals who quit smoking for a prolonged period will be similarly analyzed, and individuals who have resumed smoking will be excluded. Quitting smoking for a prolonged period is defined as, at 9 months after enrollment, indicating in an interview that one has not smoked in the past month and having a concentration of exhaled CO of 7 ppm or lower.

#### Items analyzed and analytical methods

The participant characteristics in both groups will be described statistically. Mean values for weight, abdominal circumference, changes in marker levels for glucose and lipid metabolism, along with changes in cardiovascular biomarkers from enrollment until 9 months after enrollment, will be compared between the two groups. The distribution of individual indices at baseline and at 9 months after enrollment will be determined for the two groups, and a *t* test or a Wilcoxon rank-sum test will be performed. In addition, Fisher’s exact test will compare the percentage of individuals who quit smoking for a prolonged period in the two groups. The level of significance will be 0.05 or less. A two-tailed alpha of 5% and a two-sided 95% confidence interval will be used. The statistical analysis will be performed by a statistician. The primary endpoint will be assessed statistically to facilitate an interim analysis. Nutritional guidance will be deemed effective or ineffective in the final analysis using the statistic *Z*_prop_ = 1.567. An interim analysis will be performed after the 125 participants (an intermediate number of individuals out of the total enrollment) complete follow-up. This interim analysis will determine whether to terminate this trial for efficacy or futility. Trial termination for efficacy will be based on *Z*_prop_ > 2.538, whereas trial termination for futility will be based on *Z*_prop_ < 0.698.

#### Early termination of this trial

This trial will be terminated early in any of the following situations:

(1) the trial needs to be terminated for efficacy or futility based on the results of interim analysis; (2) the trial will continue based on the results of interim analysis and the target number of participants is modified or the target number of participants is reached prior to the scheduled conclusion of enrollment; (3) the Central Ethics Review Committee recommends that the trial be terminated due to serious adverse events; or (4) the Central Ethics Review Committee deems that the trial cannot be completed due to limited enrollment.

## Discussion

Smokers are typically known to gain weight after SC [5]. Specific causes of weight gain after SC are not known; however, studies have suggested various factors, such as increased appetite, restoration of taste and smell, increased dietary intake as a result of oral cravings, improved gastric mucosal microcirculation, and a lower resting basal metabolic rate [23]. Weight gain is noted approximately 3 years after quitting smoking [24] worsening glucose tolerance [23, 25, 26]. The fear of gaining weight leads individuals to resume smoking [27, 28]. A study on women who resumed smoking due to a fear of gaining weight has reported that SC treatment with nicotine gum and a very-low-calorie diet helped to inhibit weight gain and increased the percentage of women who successfully quit smoking [13]. Drug therapy, such as nicotine replacement therapy, inhibits weight gain during SC treatment [23], leading to an increased percentage of individuals who successfully quit smoking [29]. Nonetheless, increased appetite as a result of a serotonin deficiency has been noted upon the conclusion of drug therapy [30]. This will be the first large-scale, prospective, randomized controlled trial to examine the effectiveness of nutritional guidance for individuals who are likely to gain weight after SC upon the conclusion of treatment.

The primary endpoint in this trial is the adiponectin level. Adiponectin has anti-inflammatory action, action to protect blood vessels, and action to inhibit atherosclerosis. Adiponectin levels in the blood decrease as a result of obesity, particularly because of increased visceral fat [31]. Serum adiponectin levels are also known to decrease because of smoking [31], which might be closely related to the occurrence of cardiovascular events [32, 33]. A study has reported that adiponectin levels predict future cardiovascular disease [34–36]. If weight gain is inhibited among individuals who quit smoking for a prolonged period, then their cardiovascular risk might decrease. In fact, our group has previously reported that patients who quit smoking for a prolonged period and whose weight gain was inhibited showed improved levels of cardiovascular biomarkers, such as increased serum adiponectin levels. Given that adiponectin is a marker that reflects both the effects of quitting smoking and inhibition of weight gain, adiponectin level was chosen as the primary endpoint for this trial.

The current trial will determine the effectiveness with which nutritional guidance prevents obesity after SC and will compare the effects of nutritional guidance on cardiovascular, glucose, and lipid metabolism biomarkers. This trial will involve individuals who successfully quit smoking with the help of a SC clinic and gained weight. If the advantages of quitting smoking are enhanced further by preventing obesity after quitting smoking, then cardiovascular disease development, and subsequent medical expenditures, should be sharply reduced. An increased appetite after quitting smoking is a sign that nicotine withdrawal is abating, but the undeniable reality is that forcefully curtailing appetite through severe dietary restrictions can prompt individuals to resume smoking. Nutritional guidance can inhibit weight gain, but it does more harm than good if the individual resumes smoking. Thus, the current trial will also examine the effects of nutritional guidance on the percentage of individuals who quit smoking for a prolonged period. Treatment in this trial is completely covered by National Health Insurance. However, the participants receiving nutritional guidance will be put at a disadvantage because of the increased number of visits and the fee for nutritional guidance. Nonetheless, a study has reported that nutritional guidance after quitting smoking helps inhibit obesity after SC and increases the percentage of individuals who quit smoking for a prolonged period [12]. Nutritional guidance could, potentially, reduce an individual’s cardiovascular risk. The results of this trial should help to improve the quality of SC treatment in the future.

### Trial status

At the time of manuscript submission, recruitment for this study is ongoing.

## Additional file

Additional file 1: Standard Protocol Items: Recommendations for Interventional Trials (SPIRIT) Checklist. (DOC 121 kb)

## Abbreviations

AT-LDL: α1-antitrypsin low-density lipoprotein complex
BMI: Body Mass Index
CO: Carbon monoxide
EDC: Electronic Data Capture
FTND: Fagerström Test for Nicotine Dependence
hsCRP: High-sensitivity C-reactive protein
LOX-1: Leptin-like oxidized low-density lipoprotein receptor-1
SAA-LDL: Serum amyloid A-LDL complex
SC: Smoking cessation
SD: Standard deviation
SDS: Self-rating Depression Scale
